# Optimization of the enzyme‐assisted aqueous extraction of phenolic compounds from pistachio green hull

**DOI:** 10.1002/fsn3.900

**Published:** 2018-12-03

**Authors:** Amir Pouya Ghandahari Yazdi, Mohsen Barzegar, Mohammad Ali Sahari, Hassan Ahmadi Gavlighi

**Affiliations:** ^1^ Department of Food Science and Technology Tarbiat Modares University Tehran Iran

**Keywords:** cellulase, enzymatic extraction, pectinase, pistachio green hull, tannase

## Abstract

Phenolic compounds form an essential part of the human diet because of their functional properties. In this study, the extraction conditions of phenolic compounds from pistachio green hull were optimized by enzymatic method (using pectinase, cellulase, and tannase enzymes). For this purpose, the effective factors including the solid to solvent ratio, enzyme concentration, particles size, and extraction time were optimized. Also, the effect of enzymatic extraction on the antioxidant activity of the extracts were investigated using three different methods (DPPH˙, ABTS˙^+^, and FRAP). The profile of phenolic compounds was determined using HPLC/DAD. The results showed that all the studied enzymes were significantly effective in increasing the extraction efficiency. The combination of cellulase, pectinase, and tannase enzymes under their optimal conditions increased the extraction yield up to 112% in comparison with the solvent extraction method. The results of three antioxidant tests showed that the antioxidant properties of the enzymatic extracted compounds increased significantly compared to the control sample (compounds extracted by the solvent method). The DPPH˙ test results indicated that the antioxidant property of the enzymatic extracted compounds was 71% more than the control extract. The different enzymes changed the phenolic compounds’ profile so that the pectinase and cellulase enzymes increased the amount of phloroglucinol (more than three times) and decreased the amount of gallic acid (more than 4.5 times) in comparison. In addition, tannase and its combination with other enzymes increased the gallic acid content by 2.6‐fold and 4.6‐fold compared to the control sample, respectively.

## 1 INTRODUCTION

Nowadays, in order to avoid the side effects of chemical ingredients in foodstuffs, researchers try to replace them with natural ingredients. Several attempts have been made to find natural antioxidants from plant sources (Ghaderi‐Ghahfarokhi, Barzegar, Sahari, & Azizi, 2016; Roostaee, Barzegar, Sahari, & Rafiee, 2017), mainly due to safety issues, as well as the toxicity of synthetic antioxidants. For instance, it has been shown that some commonly used synthetic antioxidants such as butylated hydroxy anisole (BHA), tert‐butyl hydroquinone (TBHQ), and propyl gallate can act as tumor creator or tumor developer in laboratory animals or damage the DNA in the presence of metal ions like Fe and Cu (Dolatabadi & Kashanian, 2010). Among the natural antioxidants are phenolic compounds that are found in different parts of plants. These compounds protect the body with strong antioxidant activity against the oxidation and cellular damage caused by free radicals (Shilpi, Shivhare, & Basu, 2013).

Pistachio with the scientific name of *Pistacia vera* L. is from the Anacardiaceae or pistachios family. Iran is one of the main producers and exporters of pistachio in the world. Pistachio production of Iran was about 261,000 tons (Ahmadi et al., 2016). Goli, Barzegar, and Sahari (2005) showed that PGH extract contains significant amounts of phenolic compounds, which is considerable compared to other sources. Its antimicrobial and antioxidant properties have also been proven in other researches (Goli et al., 2005; Rajaei, Barzegar, Mobarez, Sahari, & Esfahani, 2010).

The original phenolic compounds of PGH vary according to the variety and solvent used for extraction. Some PGH compounds include gallic acid, 4‐hydroxybenzoic acid, protocatechuic acid, eriodictyol‐7‐O‐glucoside, isorhamnetin‐7‐O‐glucoside, quercetin‐3‐O‐rutinoside, isorhamnetin‐3‐O‐glucoside naringin, naringenin, catechin, epicatechin, and luteolin (Barreca et al., 2016; Lalegani, Ahmadi Gavlighi, Azizi, & Sarteshnizi, 2018). Up to now, several researches have been showed the properties of PGH extracts (Abolhasani, Barzegar, & Sahari, 2018; Rafiee, Barzegar, Sahari, & Maherani, 2017; Roostaee et al., 2017). Nanoliposomal carriers for phenolic compounds of PGH extracts were studied by Rafiee et al. (2017). They reported that nanoliposomes were composed of 1% lecithin with 1000 ppm of phenolic compounds. The antioxidant properties of nanoliposomes in soybean oil have also been studied. The results showed that the PGH free extract had higher antioxidant activity than the encapsulated one; however nanoliposomes improved the solubility of phenolic compounds, and gradually released the above compounds and stability (Roostaee et al., 2017). Abolhasani et al. (2018) indicated the enhancement of phenolic compounds’ extraction yield, as well as the antioxidant and antityrosinase properties of PGH extract by gamma irradiation. Due to the fact that pistachio is massively produced in Iran, the amount of skin produced is high, which can be used as a low cost but rich source of phenolic compounds. Extraction is a critical first step in commencement of separating various bioactive compounds from plant materials. But the extraction yield of bioactive compounds is low due to the presence of complex cell wall polysaccharides, such as alginate and carrageenan. The high content of various polysaccharides present in the cell wall impedes the access to bioactive compounds. Generally, cell walls are formed of complex biopolymers like cellulose, hemicellulose, lignin, and pectin (Doi & Kosugi, 2004). Due to low density of bioactive compounds, low efficiency of the solvents used to extract these compounds, high energy, high durability, the residue of solvents in the extracts, and the decline of the quality of the final product, and environmental problems, today, modern methods of extraction of these compounds have been considered by researchers (Yang, Jiang, Shi, Chen, & Ashraf, 2011).

Enzymes have been used in many aquatic environments according to the characteristics of cell membrane hydrolysis as well as the catalytic activity and performance under mild conditions. Enzymes reduce solvent consumption and increase the extraction efficiency of bioactive compounds. Many studies have been conducted on enzyme‐derived extraction; commercial enzymes are also available in this field. Enzymes have been used specifically to treat plant materials before the traditional techniques of extraction (Yang et al., 2011). They have been used in numerous researches to extract phenolic compounds from plants and fruits including grapes, green tea, grapefruit, tomatoes, and apples (Fernández, Vega, & Aspé, 2015; Kammerer, Claus, Schieber, & Carle, 2005; Neagu, Leopold, Thonart, Destain, & Socaciu, 2014; Pinelo, Zornoza, & Meyer, 2008). Increasing the efficiency of extraction of phenolic compounds by enzymes has been reported in different studies; for example, Li, Smith, & Hossain, 2006 investigated the extraction of phenolic compounds from citrus peel and compared the obtained extracts’ antioxidant properties. The extraction of luteolin and apigenin by the enzymes of cellulase, beta‐glucosidase, and pectinase from pigeon pea has also been studied. The results showed that pectinase was more effective in the extraction of the mentioned compounds such that under optimum conditions, the amount of apigenin and luteolin increased by 239% and 248%, respectively (Fu et al., 2008). The extraction of phenolic compounds from red algae *Palmaria palmata* was done by several commercial enzymes of proteases and carbohydrases (Wang et al., 2010). In a similar research, phenolic compounds extracted from green tea were examined by several commercial enzymes, and tannase was used to increase the extracts’ antioxidants properties. The total phenolic compounds, flavonoids, and DPPH radical inhibitory properties were significantly higher than those of other treatments extracted with Viscozyme (Hong et al., 2013). Fernández et al. (2015) evaluated the effects of pectinase, cellulase, and tannase enzymes on the extraction of phenolic compounds from the skins and seeds of grape. The results revealed that pectinase had the most effect on the extraction efficiency of phenolic compounds such that it increased the amount of phenolic compounds by 2.5 times compared to the control sample.

The extraction of phenolic compounds of PGH has been investigated by various solvents, and it has been indicated that the best solvent for extraction of the phenolic compounds of PGH is water (Rajaei, Barzegar, Mobarez, et al., 2010). In addition, the extraction of the phenolic compounds of PGH has been optimized using ultrasound, microwave, and maceration processes. The results showed that the extraction yield through ultrasound was higher than by maceration and microwave methods (Rajaei, Barzegar, Hamidi, & Sahari, 2010). Based on the problems associated with the solvent extraction of bioactive compounds, enzymes were used in this research to extract phenolic compounds from PGH.

The aims of our study are as follows: (a) to optimize the enzymatic extraction conditions of phenolic compounds of PGH with the three enzymes of tannase, cellulase, and pectinase (particle size, amount of enzyme, time, and solvent to solid ratio); (b) to determine the antioxidant properties of extracted compounds; and (c) to investigate the impact of enzymatic extraction on the phenolic compounds profiles of samples by HPLC/DAD method.

## 2 MATERIALS AND METHODS

### 2.1 Plant material

Pistachio green hull (*Ahmadaghaei* variety) was prepared from the Agricultural Research Centre of Yazd (Yazd Province, Iran). After drying (in shadow and at 35°C), the samples were milled and then sieved. The samples used for the extraction step were separated by meshes 18, 30, and 40. Then they were kept in a freezer at −20°C until use.

### 2.2 Chemicals

Folin–Ciocalteu, 2, 2‐diphenyl‐1‐picrylhydrazyl (DPPH), 2, 2′‐azino‐bis (3‐ethylbenzothiazoline‐6‐sulfonic acid) diammonium salt (ABTS), gallic acid, phloroglucinol, 3, 5‐dinitrosalicylic acid, protocatechuic acid, 4‐hydroxybenzoic acid, vanillic acid and potassium persulphate were purchased from Sigma (St. Louis, MO, USA). 2, 4, 6‐tris (2‐pyridyl)‐s‐triazine (TPTZ), acetic acid, and methanol were obtained from the Merck Chemical Co. (Darmstadt, Germany). Pectinex BE color enzyme from Novozymes Ferment (Bagsvaerd, Denmark), cellulase enzyme from Sigma, and tannase enzyme were provided by Kikkoman Biochemifa (Japan, Tokyo). The characteristics of the studied enzymes are shown in Table 1.

**Table 1:** Characteristics of the enzymes used in this study

Enzyme	Optimum conditions			Source
	Activity	pH	*T* (°C)	
Pectinex BE Color (P)	3800 ≥ U/ml	4.0	40	*A. niger*,* A. aculeatus*
Cellulase (C)	2500 ≥ U/ml	5.0	37	*Trichoderma reesei*
Tannase (T)	500 ≥ U/g	5.0–5.5	40	*A. oryzae*

### 2.3 The enzymatic extraction of phenolic compounds from PGH

The enzymatic extraction was done according to the method of Fernández et al. (2015) with briefly modifications. The selected hydrolysis conditions were based on the temperature and pH activity curves of each enzyme, as given on the enzyme suppliers’ data sheets (Table 1). For this purpose, 0.4 g PGH powder was placed in a 50 ml falcon. Then, 40 ml of enzymatic solution was added at a specific concentration, and the extraction was carried out under the optimum conditions of each enzyme (temperature and pH). Sodium acetate buffer (50 mM) was used as a solvent and to maintain the optimal pH conditions. The enzymatic extraction of PGH was accomplished in a thermostatically controlled orbital shaker (IKA, KS 4000 i control, Germany) with gentle agitation (150 rpm) in the dark. At the end of each extraction, the sample was centrifuged at 4°C for 10 min at 2500 *g*. Finally, the supernatant was filtered using Whatman No. 41 filter paper and kept in a brown flask at −20°C until analysis. In this study, the effect of four variables, that is, time (1, 2, 3, 4, and 5 h), solid to solvent ratio (1:20, 1:40, 1:80, 1:100, and 1:150 g/ml), particle size (≤0.40, between 0.60–0.40, 0.60–1.00, and ≥1.00 mm) and concentration of pectinase (P) (0, 1.9, 3.8, and 7.6 U/ml), cellulase (C) (0, 1.25, 2.5, and 5 U/ml) and tannase (T) (0, 0.1, 2, and 4 U/g) was evaluated using one‐factor‐at‐a‐time statistical method. After optimizing the extraction conditions with each enzyme separately, the enzymatic extraction was performed by different combinations of enzymes: (cellulase + pectinase (CP), cellulase + tannase (CT), pectinase + tannase (PT), and finally, cellulase + pectinase + tannase (CPT)).

### 2.4 Determination of total phenolic compounds (TPC)

The Folin–Ciocalteu method was used in order to determine the TPC of extracts according to the method of Slinkard and Singleton (1977) and the amount of phenolic compounds was reported as mg gallic acid equivalent per 100 g of dry pistachio green powder (mg GAE/100 gdw).

### 2.5 Antioxidant activity assessments

#### 2.5.1 DPPH˙ assay

DPPH radical scavenging activity was determined according to the method of Hatano, Kagawa, Yasuhara, and Okuda (1988). The radical scavenging activity (RSA) of the extract was determined according to formula reported by Abolhasani et al., 2018. An IC_50_ factor was used to evaluate the antioxidant activity.

#### 2.5.2 ABTS˙^+^ assay

The ABTS radical cation test was performed in accordance with the method of Re et al. (1999). The radical activity of the extract was determined according to the method of Lee, Lee, Gal, Moon, and Park (2006).

#### 2.5.3 FRAP assay

The FRAP test was performed in accordance with Benzie and Strain (1996). The antioxidant power expressed as the concentration of antioxidants having a ferric reducing ability equivalent to that of 1 μM FeSO_4_.

### 2.6 Separation and determination of the phenolic compounds of PGH extract using HPLC/DAD

Separation and determination of the phenolic compounds of extracts were done by using the Azura (Knauer, Berlin, Germany) HPLC device equipped with UV‐Vis photodiode array detector (DAD 2. one langmuir, Knauer), LC pump (P 6.1L), and a Prodigy reverse phase column (250 × 4.6 mm, particle size 5 μm, Phenomenex, USA) at a wavelength of 700–190 nm according to the method of Barreca et al. (2016). The retention times of the standard compounds were compared with those of the PGH extracted compounds in order to identify phenolic compounds. The amounts of separated phenolic compounds were determined by the external standard method. Each experiment was performed in triplicates.

### 2.7 Statistical analysis

All tests were done in triplicates, and the mean ± *SD* (*M* ± SD) of the data were reported. LSD test was used to evaluate the presence of significant differences at 95% confidence level. For this purpose, statistical analysis was accomplished using SAS 9 software and drawing the related charts using Excel 2013 software.

## 3 RESULTS AND DISCUSSION

### 3.1 Effect of different enzyme concentrations on extraction of phenolic compounds

Optimization of the concentration of pectinase, cellulase, and tannase enzymes was done with regard to the optimum conditions of each enzyme (Table 1). For this purpose, other factors such as solid/liquid ratio (1:100 mg/ml), time (3 h), and particle size (0.4–0.595 mm) were kept constant, and the concentrations of pectinase, cellulase, and tannase over the ranges of (0, 1.9, 3.8, and 7.6 U/ml), (0, 1.25, 2.5, and 5 U/ml), and (0, 1.0, 0.2, and 4 U/g), respectively, were optimized.

#### 3.1.1 Pectinase

The effect of various concentrations of pectinase on the extraction yield of PGHs’ phenolic compounds showed that pectinase was very effective in extracting these compounds such that, there was an increase in the amount of extraction of phenolic compounds by 42% compared to the control sample by adding 3.8 U/ml of pectinase. Adding more enzyme (>3.8 U/ml), the extraction yield increased only 5%. Because of economic issues, we selected it as an optimum enzyme concentration. Increased extraction yield of bioactive compounds from various sources like pectinase and cellulose has been reported (Choudhari & Ananthanarayan, 2007; Dzogbefia, Ofosu, & Oldham, 2008; Wilkins, Widmer, Grohmann, & Cameron, 2007). Fernández et al. (2015) showed a 2.5‐fold increase in the extraction yield of phenolic compounds from the skin and seed of grapes by using pectinase in comparison with the control. The results indicated no significant difference between the 1% and 5% concentrations of enzyme to substrate ratio, but this difference was significant in the 10% concentration of enzyme to substrate ratio. In contrary, Chamorro, Viveros, Alvarez, Vega, and Brenes (2012) showed that pectinase had no significant effect on the extraction yield of phenolic compounds from grapes’ pomace and seed.

Due to the fact that the cell wall consists of cellulose, hemicellulose, pectin, and protein, as well as phenolic compounds linked to available polysaccharides with hydrogen and hydrophobic bonds, various enzymes such as cellulases, pectinases and hemicellulases can be used as hydrolyzing agents to destroy the cell wall structure. These enzymes can also be used to increase the cell walls’ penetration potential, which results in the release of phenolic compounds and increasing the extraction yield of bioactive compounds (Miron, Herrero, & Ibáñez, 2013). Another mechanism is probably the direct action of the enzyme on the breakdown of esters’ or ethers’ linkages between the phenols and the plant cell wall polymers (Pinelo et al., 2008). In fact, pectinase causes pectin to be displaced or broken, leading to the destruction of the cell wall and facilitating the release of phenolic compounds (Fernández et al., 2015).

#### 3.1.2 Cellulase

The effect of different concentrations of cellulase on the phenolic compounds’ extraction yield was similar to those in Section 3.1.1. Adding 2.5 U/ml of the enzyme increased the extraction yield by about 22%, while doubling the enzyme concentration had no significant effect on the extraction yield (*p* < 0.05). According to the obtained results, the best concentration of cellulase was 2.5 U/ml. Similar results were reported by Fernández et al. (2015), but the findings of Chamorro et al. (2012) indicated that cellulase had no effect on increasing the extraction yield of the phenolic compounds of grape seed and pomace.

Cellulase affects the cellulose existing under the main layer and the midline lamella of the plant cells. The main layer consists of a rigid and strong skeleton of cellulose, which is located in a gel‐like matrix of hemicellulose, pectic, and glycoprotein. Cellulase accelerates the breakdown of cellulose into glucose and cellobiose (Choudhari & Ananthanarayan, 2007). In fact, it accelerates the hydrolysis of endo‐1, 4‐ß‐d‐glycosidic bond in cellulose, as well as the conversion of cello‐oligosaccharide cellotriose to cellohexaose.

#### 3.1.3 Tannase

The effect of tannase on the extraction yield of phenolic compounds showed that increasing of tannase concentration up to 4 U/g increased the extraction yield of phenolic compounds (up to 51%). The 4 U/g concentration was selected as the optimum concentration because higher concentrations of the enzyme were not economically feasible. Increasing the extraction yield of phenolic compounds by using tannase has been reported in other studies as well (Chamorro et al., 2012; Fernández et al., 2015; Martins, Roberto, Blumberg, Chen, & Macedo, 2016). Chamorro et al. (2012) reported that the extraction yield of phenolic compounds from grape seed increased by up to 41% by using tannase. Also Fernández et al. (2015) revealed that tannase increased the extraction yield of phenolic compounds from grape skin by 1.29‐fold.

Tannase has esterase and depsidase activities (Farias, Gorbea, Elkins, & Griffin, 1994). Tannase causes complete hydrolysis of tannic acid and converts it into gallic acid and glucose. Intermediate products in this reaction include 1, 2, 3, 4, 6‐pentagalloyl glucose, 6, 4, 3, 2‐tetragalloyl glucose, and two types of mono‐galloyl glucose (Iibuchi, Minoda, & Yamada, 1972). Tannase affects the gallotannine ester linkages, as well as the gallotannins, ellagitannins, and complexed tannins; however it has no effect on the condensed tannins. This enzyme is capable of breaking the ester linkages but it does not have the ability to break the carbon–carbon bonding. Due to the mentioned mechanism, the tannase enzyme increases the amount of phenolic compounds (Haslam & Stangroom, 1966).

### 3.2 Effect of solid to solvent ratio on the extraction yield of phenolic compounds

To optimize the solid to solvent ratio, the optimum concentration of each enzyme (pectinase: 3.8 U/ml, cellulase: 2.5 U/ml and tannase: 4 U/g) was used, and all factors were considered constant except the solid to solvent ratio (time: 3 h, particle size: 0.40–0.60 mm). The effect of solid to solvent ratio on the extraction yield of phenolic compounds was investigated at five different levels (1:20, 1:40, 1:80, 1:100, and 1:150 g/ml). This parameter had significant effect on the extraction yield. Increase of the solid to solvent ratio from 1:20 to 1:80 increased the extraction yield of phenolic compounds about, 25%, 63%, and 97% by using pectinase, cellulase, and tannase, respectively. Similar results involving the effect of the solid to solvent ratio on the extraction yield of phenolic compounds have previously been reported by other researchers (Cacace & Mazza, 2003; Fernández et al., 2015). Fernández et al. (2015) reported that the amount of extracted phenolic compounds from grape skin in the ratio of 1:100 g/ml (solid to liquid) was greater than in the ratio of 1:200 g/ml. These results are consistent with the principles of mass transfer. Accordingly, the driving force during the mass transfer is the concentration gradient between the solid and the bulk of the liquid, which is more when a lower solid to solvent ratio is used (Cacace & Mazza, 2003). It is to be noted that any increase in the solvent to solid ratio reduces the extraction efficiency. Increasing the solid to solvent ratio from 1:80 to 1:150 decreased the extraction yield of phenolic compounds about 10%, 41%, and 24% for the treatments containing pectinase, cellulose, and tannase, respectively. However, it seems that the amount of extracted phenolic compounds remained constant. Based on our results, the ratio of 1:80 g/ml was selected as an optimum ratio for extraction of phenolic compounds from PGH.

### 3.3 Effect of particle size on the extraction yield of phenolic compounds

The effect of different particle sizes (≤0.40, 0.40–0.60, 0.60–1.00, and ≥1.00 mm) at constant enzyme concentrations (pectinase, 3.8 U/ml; cellulase, 2.5 U/ml; tannase, 4 U/g), solid to solvent ratio (1:80), and time (3 h) was studied on the extraction yield. Decreasing the particle size of PGHs increased the extraction yield of phenolic compounds. The results showed that reducing the particle size from 1.00 to 0.40 mm increased the extraction yield of phenolic compounds in the presence of the mentioned enzymes by more than two times. Increasing the extraction of bioactive compounds has been reported by reducing the particle size (Landbo & Meyer, 2001; Ranveer, Patil, & Sahoo, 2013). Landbo and Meyer (2001) indicated that a decrease in the particle size of black currant pomace from 500–1000 μm to <125 μm increased the phenolic extraction yields 1.6–5.0 times. Ranveer et al. (2013) showed that the smaller particle size of tomato processing waste improved the extraction yield of lycopene. The particle size reduction of plant material increases the extraction yield because it may cause ruptures of the cell walls, and milling increases the surface area of the powder, and therefore, increases the contact surface of solvent and sample (Pinelo, Tress, Pedersen, Arnous, & Meyer, 2007). According to our results, the particle sizes smaller than 0.40 mm were chosen for extraction of phenolic compounds from PGH.

### 3.4 Effect of time on extraction yield of phenolic compounds

This experiment investigated the effect of different times (1, 2, 3, 4, and 5 h) on the extraction yield of phenolic compounds from PGH at other constant conditions (enzyme concentration: pectinase, 3.8 U/ml, cellulase, 2.5 U/ml, tannase, 4 U/g), solid to solvent ratio (1:80) and particle size ˂0.40 mm. According to the obtained results, the extraction yield of phenolic compounds increased (by 2.15‐, 2.20‐, and 1.70‐fold by pectinase, cellulase, and tannase, respectively) up to 4 h and after that remained constant. Therefore, in other experiments, 4 h was selected as an optimum extraction time. Similar trends have been published by others (Fu et al., 2008; Ranveer et al., 2013). Fu et al. (2008) reported that the highest concentration of luteolin and apigenin was observed after 18 h and after that the extraction yields remained almost constant. In another study, the highest recovery of lycopene from tomato processing waste was achieved for 4 h duration by pectinase and cellulase (Ranveer et al., 2013). As a result, 4 h was selected as the optimum time for extraction of phenolic compounds from PGH.

### 3.5 Combinations of enzymes

**Figure 1:**
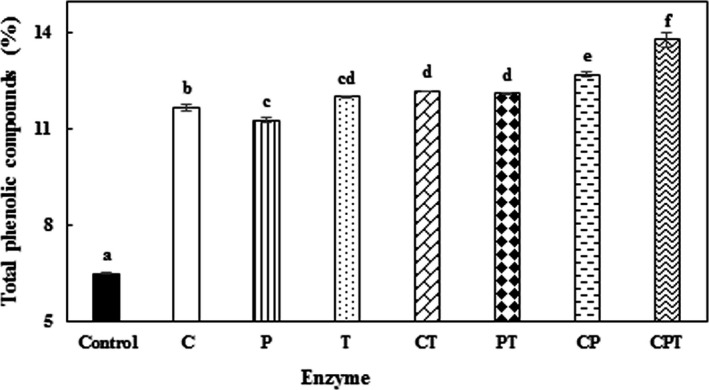
Effect of enzymes’ combination on the extraction yield of the phenolic compounds of pistachio green hull. CT (cellulase + tannase), PT (pectinase + tannase), and CPT (cellulase + pectinase + tannase). Conditions: concentration of pectinase, cellulase, and tannase: 3.18 U/ml, 2.5 U/ml, and 4 U/g, respectively, particle size ˂0.40 mm, and solid to solvent ratio 1:80. Data are means ± *SD* (*n* = 3). Values with different lowercase letters are significantly different (LSD,* p* < 0.05)

Finally, combinations of the studied enzymes (cellulase + pectinase (CP), cellulase + tannase (CT), and pectinase + tannase (PT) and cellulose + pectinase + tannase (CPT)) were used to extract phenolic compounds from PGH. For this purpose, optimized parameters (solid to solvent ratio (1:80), time (4 h), enzyme concentration (pectinase: 3.18 U/ml, cellulose: 2.5 U/ml and tannase: 4 U/g), and particle size (˂0.40 mm)) were used. Also, based on the pretest results, 37°C and pH = 4.0 were selected for extraction of phenolic compounds by CPT. According to Figure 1, using the mixture of enzymes increased the extraction yield of phenolic compounds. The results further showed that CT, PT, CP, and CPT increased the extraction yield of phenolic compounds by 88%, 87%, 96%, and 112% compared to the control sample, respectively. The extraction yield of phenolic compounds obtained with the combination of enzymes from PGH was more than that obtained by the individual enzymes. The combinations of enzymes increased the extraction yield of phenolic compounds by 23%, 19%, and 15% compared to pectinase, cellulase, and tannase, respectively. Similar results have been reported by Pinelo et al. (2008) and Chamorro et al. (2012). While Fernández et al. (2015) mentioned that the concentration of phenolic compounds extracted with the enzymatic blend from grape seeds and skins was similar to that extracted by the enzymes individually. Also Martins et al. (2016) suggested that tannase was effective in increasing the extraction yield of phenolic compounds, while PC had no effect on the extraction of phenolic compounds. CPT increased the total phenol content but the extraction yield was lower when tannase was used alone. According to our results, when the combination of the mentioned enzymes was used, more complete breakdown was achieved (Chamorro et al., 2012) and more phenolic compounds were extracted.

### 3.6 Antioxidant activity of samples

#### 3.6.1 DPPH˙ test

**Figure 2:**
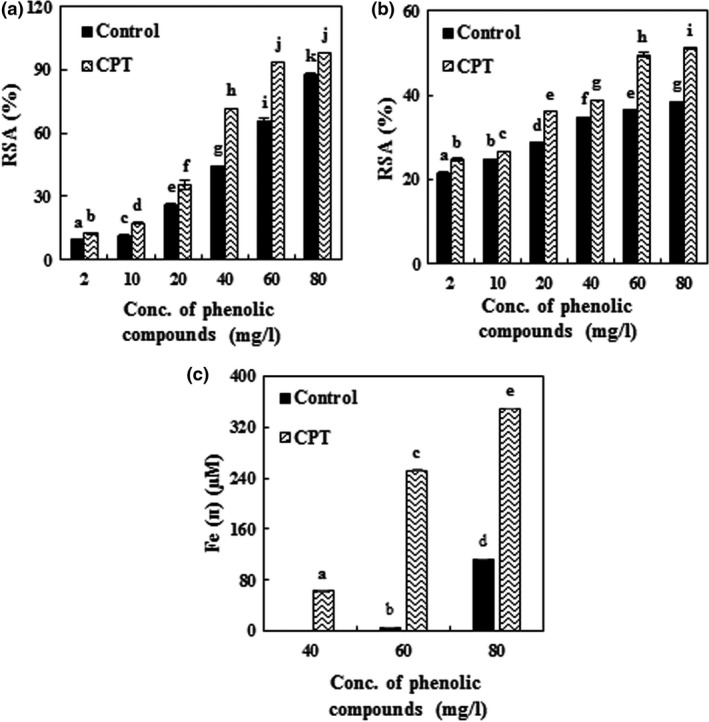
Effect of the enzymatic extraction of phenolic compounds from pistachio green hull on antioxidant activity: (a) (DPPH˙), (b) (ABTS˙^+^), (c) (FRAP). Data are means ± *SD* (*n* = 3); CPT (cellulase + pectinase + tannase); RSA, radical scavenging activity; values with different lowercase letters are significantly different (LSD,* p* < 0.05)

In the present study, by given in Section 3.5, the antioxidant activity of phenolic compounds extracted by CPT was confirmed. Figure 2a shows the free radical scavenging potential of the PGH extract toward DPPH. As indicated, there is a linear and positive correlation between the concentration of phenolic compounds and their antioxidant activity because of their hydrogen donating abilities (Pyo, Lee, Logendra, & Rosen, 2004; Robards, Prenzler, Tucker, Swatsitang, & Glover, 1999). Enzymatic hydrolysis of PGH extract by CPT significantly increased the radical scavenging capacity (EC_50_ = 26.59 mg/l), which was 70.3% more than that of the control (EC_50_ = 45.29 mg/l). Increased antioxidant activity after enzymatic hydrolysis has been reported in similar studies (Chamorro et al., 2012; Wang et al., 2010). Chamorro et al. (2012) found that after the enzymatic hydrolysis of grape seed extract by tannase, its radical scavenging capacity was increased (4%). Also, the antioxidant activity of grape pomace treated with Pektozyme, tannase, and combination of them increased by up to 12%, 20%, and 32%, respectively.

At higher concentrations of phenolic compounds, due to the increased number of hydroxyl groups in the reaction medium, the possibility of hydrogen donation to free radicals increases, which leads to increased radical scavenging capacity. The radical scavenging capacity of various extracts depends greatly on the number and position of hydroxyl groups and the molecular weight of phenolic compounds. The lower molecular weight of phenolic compounds makes the hydroxyl groups be more readily available (Jung, Seog, Choi, Park, & Cho, 2006; Ramarathnam, Ochi, & Takeuchi, 1997; Rice‐evans, Miller, Bolwell, Bramley, & Pridham, 1995). Increased release of phenolic compounds by enzymes suggests that enzymes may have selective activities that directly liberate antioxidant phenols or change the released phenols to be more potent antioxidant compounds (Chamorro et al., 2012).

#### 3.6.2 ABTS˙^+^ test

The results of ABTS˙^+^ assay were similar to those of the DPPH˙ method (Figure 2b). As shown, the RSA has been enhanced after applying the enzymatic hydrolysis of PGH by CPT. The RSA of the control sample and hydrolyzed compounds treatment by CPT is 38.3% and 51.1%, respectively, when the concentration of phenolic compounds is 80 mg/l. The results indicated a positive linear correlation between the concentration of phenolic compounds and their antioxidant activities. Compared to the DPPH˙ test, the RSA at lower concentrations of phenolic compounds was higher in the ABTS˙^+^ test, but by increasing the concentration of phenolic compounds, the radical scavenging capacity was reduced, which can be attributed to the faster reaction of the ABTS radical cations in compare to the free radicals of DPPH (Ruan, Zhang, & Lin, 2008).

#### 3.6.3 FRAP test

The results obtained in this section also confirmed the results of the ABTS˙^**+**^ and DPPH˙ tests. As shown in Figure 2c, the antioxidant activity of enzymatic extract increased by up to 98% in comparison with the control sample at 80 mg/l concentration of phenolic compounds. According to Figure 2c, the extract of PGH at low concentrations of phenolic compounds was not effective on ferric ions’ (Fe^3+^) reduction to ferrous ion (Fe^2+^); however, at concentrations of 60 and 40 mg/l, ferric ion was reduced to ferrous ion for the control sample and the enzymatic hydrolysis treatment, respectively. At lower concentrations of phenolic compounds, probably more time is needed for reaction with TPTZ. Some edible phenolic compounds react slowly with TPTZ‐Fe^3+^ (López‐Alarcón & Denicola, 2013). TPTZ reacts slowly with polyphenolic solutions such as caffeic, tannic, and ferulic acids, so it requires a longer reaction time (Pulido, Bravo, & Saura‐Calixto, 2000).

### 3.7 Separation and determination of phenolic compounds of the PGH extract using HPLC/DAD

**Figure 3:**
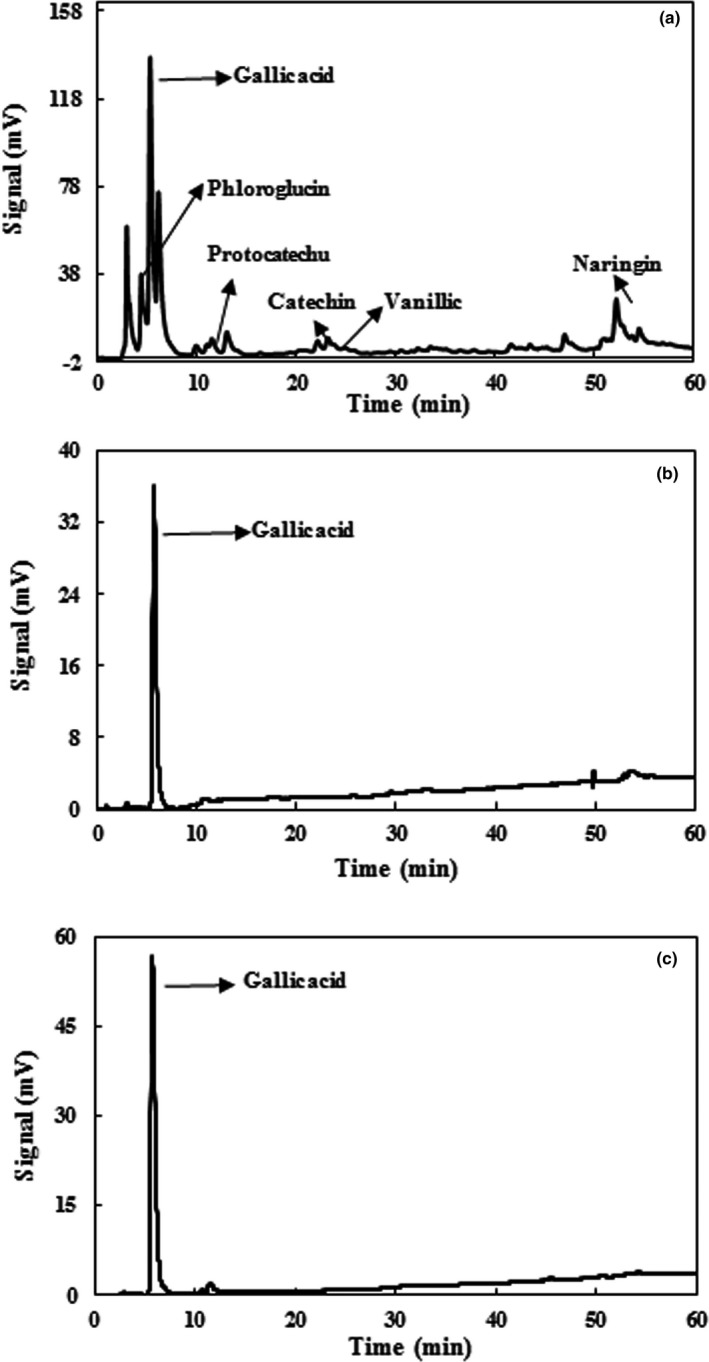
HPLC chromatogram of pistachio green hull extract: (a) non‐enzymatic extraction, (b) enzymatic extraction with tannase, (c) enzymatic extraction with cellulase + pectinase + tannase

A sample chromatogram of the phenolic compounds of the aqueous extract of PGH is shown in Figure 3a. Also, the effect of various enzymes on the type and amount of each compound is shown in Table 2. According to Figure 3, and as reported by Fattahifar, Barzegar, Ahmadi Gavlighi, and Sahari (2018), the main extracted phenolic compounds of PGH were phloroglucinol, gallic acid, naringin, vanillic acid, catechin, and protocatechuic acid. Barreca et al. (2016) found gallic acid as the most important compound extracted from PGH by methanol (50.27 ± 3.86) and ethanol (14.50 ± 0.62). As can be seen from Table 2, the effect of enzymes on the extraction of phenolic compounds has been different. For example, pectinase and cellulase caused an increase in the amount of phloroglucinol and decreased the amount of gallic acid compared to the control sample (extracted by phosphate buffer without enzymes), and the other compounds in the control sample were not detectable. Pectinase and cellulose enzymes increased the phloroglucinol content by 3.0 and 3.8 times compared to the control, respectively. As shown in Table 2 and Figure 3b and c, tannase and the combination of CPT treatment led to increase in the amount of gallic acid compared to the control sample, and other identified compounds in the control sample were not detectable in the tannase and CPT treatments. Also, tannase and the combination of CPT increased the gallic acid content by 2.6‐fold and 4.6‐fold compared to the control sample, respectively. Chamorro et al. (2012) reported that tannase treatment on grape seed extract enhanced the amount of gallic acid by 6.0 times. They found that the cellulolytic enzyme had no effect in improvement of gallic acid, whereas tannase, pectinolytic enzymes, and the combination of CPT increased the amount of gallic acid by up to 34%, 78%, and 98%, respectively. Martins et al. (2016) reported that among tannase, PC, and CPT, tannase was the most effective in increasing the extracted phenolic compounds of grape pomace. Also, the effect of tannase treatment on the increase of gallic acid in green tea has been reported by Hong et al. (2013). We guess that enzymes could destroy cell wall and also, in the acidic conditions and enzymes may bounded phenolic compounds release. Research findings show that gallic acid can be considered as the main compound responsible for the antioxidant activity of PGH extract. Gallic acid has three hydroxyl groups in its structure, leading to its high antioxidant activity. Furthermore, the most important factor influencing the antioxidant activity of phenolic compounds is their chemical structure. The ability to donate electrons or hydrogen, the formation of a complex with metals, and the antiradical activity of these compounds are related to the number and location of hydroxyl groups in the aromatic ring (Sroka & Cisowski, 2003).

**Table 2:** Phenolic compounds of solvent and enzymatic extracts of pistachio green hull^a^

Compounds	Retention time (min)	λ_max_	Non‐enzymatic extraction	Enzymatic extraction			
				Pectinase	Cellulase	Tannase	CPT^b^
Phloroglucinol	4.44	268	9.40 ± 0.05	28.82 ± 0.07	35.71 ± 0.06	n.d.	n.d.^c^
Gallic acid	5.33	268	26.20 ± 0.80	5.70 ± 0.02	4.69 ± 0.01	68.61 ± 0.71	121.10 ± 3.40
Protocatechuic acid	10.38	260	0.30 ± 0.05	n.d.	n.d.	n.d.	n.d.
Catechin	21.91	280	1.20 ± 0.02	n.d.	n.d.	n.d.	n.d.
Vanillic acid	24.41	260	1.28 ± 0.08	n.d.	n.d.	n.d.	n.d.
Naringin	52.40	280	1.40 ± 0.07	n.d.	2.21 ± 0.09	n.d.	n.d.

^a^Data are expressed as mg/g dry extract powder and they are means ± *SD* (*n* = 3); ^b^CPT: Cellulase + Pectinase + Tannase; ^c^n.d.: not detected.

## 4 CONCLUSIONS

The results of this study indicated that enzyme‐assisted extraction was effective in enhancing the extraction yield of phenolic compounds from PGH. The results revealed that combination of enzymes had a significant increasing effect on the extraction yield in comparison with using each enzyme separately. The results of DPPH˙, ABTS˙^+^, and FRAP tests showed that the phenolic compounds extracted by CPT had higher antioxidant capacity compared to the untreated samples. Based on the results of HPLC, gallic acid can be considered as the most important phenolic compound, which is highly correlated with increase in the antioxidant activity of the CPT treated PGH samples. Among the three studied enzymes, tannase showed the greatest effect on the increase of gallic acid. More studies are needed to investigate the possible use of these enzymes in the food industry. The results of this study indicated the high capacity of extraction of phenolic compounds from PGH by enzymes.

## CONFLICT OF INTEREST

There is no conflict of interest in this paper.

## ETHICAL STATEMENT

On behalf of all coauthors, I, Dr. Mohsen Barzegar, declare that this article has not been published in or is not under consideration for publication elsewhere. All authors were actively involved in the work leading to the manuscript and will hold themselves jointly and individually responsible for its content. There is no conflict of interest in this paper. Human or animal testing is unnecessary in our study.
